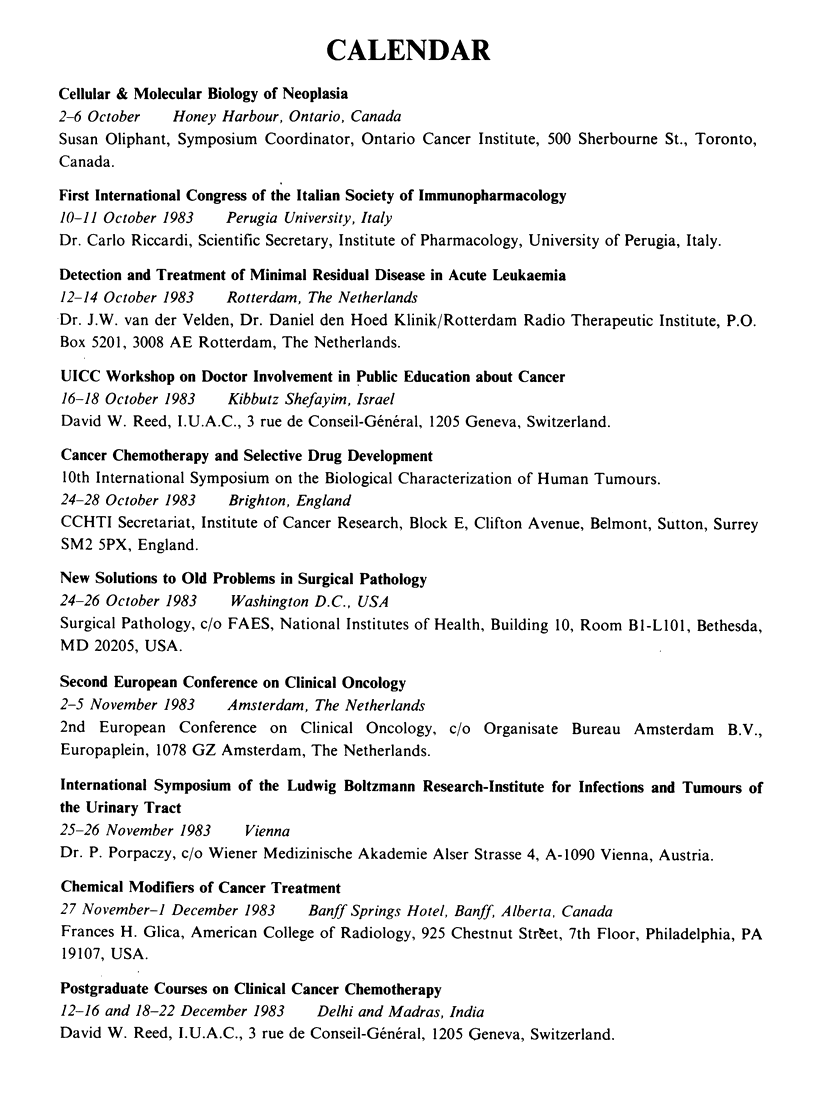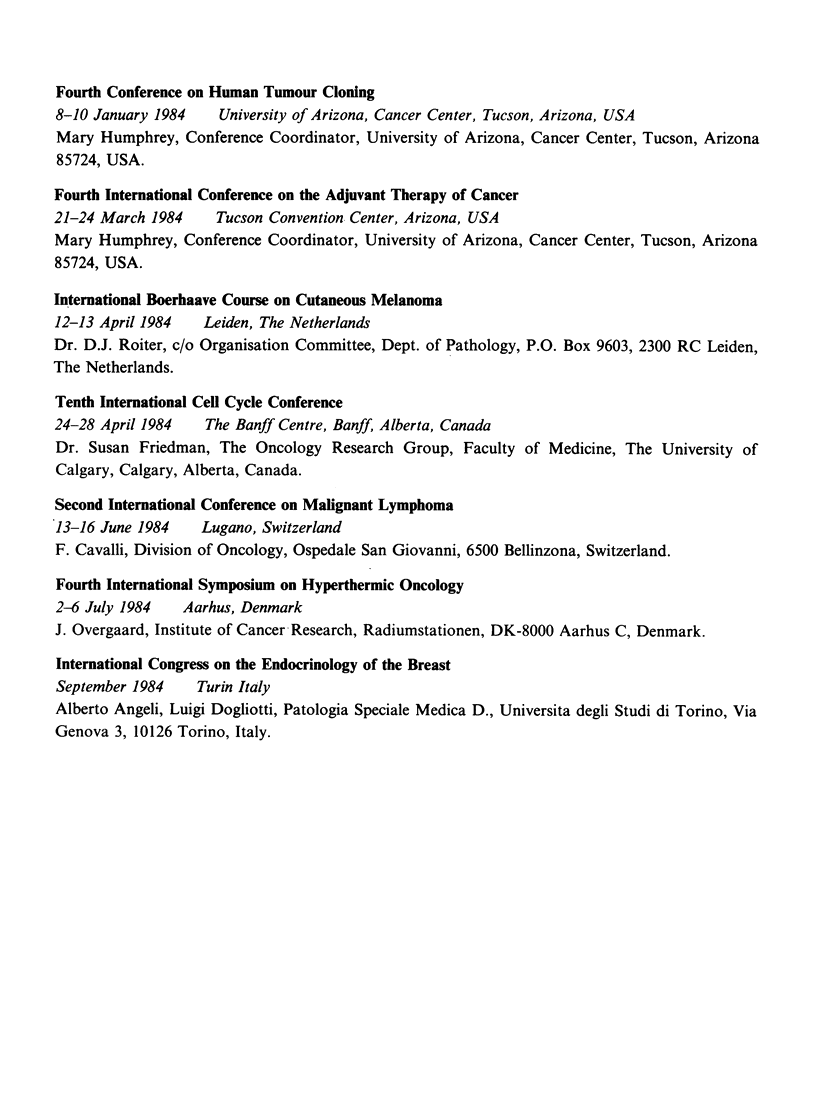# Calendar

**Published:** 1983-10

**Authors:** 


### CALENDAR

**Cellular & Molecular Biology of Neoplasia**
*2–6 October*  *Honey Harbour, Ontario, Canada*
Susan Oliphant, Symposium Coordinator, Ontario Cancer Institute, 500 Sherbourne St., Toronto, Canada.

**First International Congress of the Italian Society of Immunopharmacology**
*10–11 October 1983*  *Perugia University, Italy*
Dr. Carlo Riccardi, Scientific Secretary, Institute of Pharmacology, University of Perugia, Italy.

**Detection and Treatment of Minimal Residual Disease in Acute Leukaemia**
*12–14 October 1983*  *Rotterdam, The Netherlands*
Dr. J.W. van der Velden, Dr. Daniel den Hoed Klinik/Rotterdam Radio Therapeutic Institute, P.O. Box 5201, 3008 AE Rotterdam, The Netherlands.

**UICC Workshop on Doctor Involvement in Public Education about Cancer**
*16–18 October 1983*  *Kibbutz Shefayim, Israel*
David W. Reed, I.U.A.C., 3 rue de Conseil-Général, 1205 Geneva, Switzerland.

**Cancer Chemotherapy and Selective Drug Development**
10th International Symposium on the Biological Characterization of Human Tumours.
*24–28 October 1983*  *Brighton, England*
CCHTI Secretariat, Institute of Cancer Research, Block E, Clifton Avenue, Belmont, Sutton, Surrey SM2 5PX, England.

**New Solutions to Old Problems in Surgical Pathology**
*24–26 October 1983*  *Washington D.C., USA*
Surgical Pathology, c/o FAES, National Institutes of Health, Building 10, Room B1-L101, Bethesda, MD 20205, USA.

**Second European Conference on Clinical Oncology**
*2–5 November 1983*  *Amsterdam, The Netherlands*
2nd European Conference on Clinical Oncology, c/o Organisate Bureau Amsterdam B.V., Europaplein, 1078 GZ Amsterdam, The Netherlands.

**International Symposium of the Ludwig Boltzmann Research-Institute for Infections and Tumours of the Urinary Tract**
*25–26 November 1983*  *Vienna*
Dr. P. Porpaczy, c/o Wiener Medizinische Akademie Alser Strasse 4, A-1090 Vienna, Austria.

**Chemical Modifiers of Cancer Treatment**
*27 November–1 December 1983*  *Banff Springs Hotel, Banff, Alberta, Canada*
Frances H. Glica, American College of Radiology, 925 Chestnut Street, 7th Floor, Philadelphia, PA 19107, USA.

**Postgraduate Courses on Clinical Cancer Chemotherapy**
*12–16 and 18–22 December 1983*  *Delhi and Madras, India*
David W. Reed, I.U.A.C., 3 rue de Conseil-Général, 1205 Geneva, Switzerland.

**Fourth Conference on Human Tumour Cloning**

*8–10 January 1984  University of Arizona, Cancer Center, Tucson, Arizona, USA*

Mary Humphrey, Conference Coordinator, University of Arizona, Cancer Center, Tucson, Arizona 85724, USA.

**Fourth International Conference on the Adjuvant Therapy of Cancer**

*21–24 March 1984  Tucson Convention Center, Arizona, USA*

Mary Humphrey, Conference Coordinator, University of Arizona, Cancer Center, Tucson, Arizona 85724, USA.

**International Boerhaave Course on Cutaneous Melanoma**

*12–13 April 1984  Leiden, The Netherlands*

Dr. D.J. Roiter, c/o Organisation Committee, Dept. of Pathology, P.O. Box 9603, 2300 RC Leiden, The Netherlands.

**Tenth International Cell Cycle Conference**

*24–28 April 1984  The Banff Centre, Banff, Alberta, Canada*

Dr. Susan Friedman, The Oncology Research Group, Faculty of Medicine, The University of Calgary, Calgary, Alberta, Canada.

**Second International Conference on Malignant Lymphoma**

*13–16 June 1984  Lugano, Switzerland*

F. Cavalli, Division of Oncology, Ospedale San Giovanni, 6500 Bellinzona, Switzerland.

**Fourth International Symposium on Hyperthermic Oncology**

*2–6 July 1984  Aarhus, Denmark*

J. Overgaard, Institute of Cancer Research, Radiumstationen, DK-8000 Aarhus C, Denmark.

**International Congress on the Endocrinology of the Breast**

*September 1984  Turin Italy*

Alberto Angeli, Luigi Dogliotti, Patologia Speciale Medica D., Universita degli Studi di Torino, Via Genova 3, 10126 Torino, Italy.